# The importance of promoting a culture of academic integrity in higher education institutions in China

**DOI:** 10.1186/s41155-025-00367-w

**Published:** 2025-11-06

**Authors:** Wenwei Zhu

**Affiliations:** Research Center for Party Building in the New Era, Party School of the Qinghai Provincial Committee of C.P.C, No. 2 Huanghe Road, Xining, 810000 China

**Keywords:** Academic ethics, Educational policy reform, Fraud prevention, Higher education in China, Integrity-focused intervention, Moral development of students

## Abstract

**Introduction:**

Academic misconduct remains a pervasive issue in higher education institutions, undermining both academic integrity and the quality of the educational environment. Existing research primarily focuses on describing the forms and frequency of violations.

**Objective:**

This study evaluated the effectiveness of an educational intervention designed to modify students’ attitudes and behaviors regarding academic integrity.

**Methods:**

The study implemented a controlled experimental design with pre-test and post-test measurements. The participants were 400 undergraduate students from a Chinese university (202 males and 198 females, mean age = 21 years), who were assigned to either an experimental or control group. The six-month intervention was an educational program comprising lectures, role-playing exercises, discussions, and interactive workshops. The scale of academic dishonesty developed by Rawwas and Isakson was used to measure aspects such as acceptance of cheating, unfair advantages, data fabrication, and disregard for dishonest practices.

**Results:**

The analysis revealed statistically significant improvements across all categories among students in the experimental group after completing the program. No significant changes were observed in the control group.

**Conclusion:**

The findings confirm the efficacy of structured educational programs in reducing the propensity for academic misconduct. This study provides empirical evidence useful for universities seeking to implement ethical standards and foster a culture of academic integrity.

## Introduction

Academic integrity is a fundamental principle underpinning the credibility and functioning of higher education institutions worldwide (Holden et al., 2021). It involves upholding values such as fairness, respect, responsibility, and honesty in all academic work (Ayodele et al., 2019). Breaches of academic integrity, such as cheating, plagiarism, and fabrication, pose serious threats to the quality of learning and the reputation of institutions (Chen & Macfarlane, 2024). In the present study, academic integrity is defined as a set of established ethical norms governing student behavior in academic settings. These norms include the prohibition of cheating, data fabrication, gaining unfair advantages, and willful disregard for dishonest practices. In recent years, the rapid expansion of higher education in China has brought with it a rise in reported cases of academic dishonesty (Chen, 2022). Such incidents compromise the integrity of academic institutions and underscore the need to develop strategies that promote ethical conduct (Cotton et al., 2024). One such strategy is the implementation of educational programs explicitly designed to enhance students' understanding and internalization of academic integrity. These programs often include lectures, workshops, and role-playing activities that aim to inform students about academic expectations and the consequences of dishonest behavior (Cronan et al., 2018; Kornytska, 2023). Although such interventions have shown promise in other contexts, there is a lack of empirical evidence about their effectiveness in China—a country with unique cultural and institutional characteristics (Du, 2018). To address this gap, the present study evaluates the impact of a structured educational program on students' attitudes and behaviors related to academic integrity at a Chinese university. The study employs a controlled experimental design, comparing a group of undergraduate students who received the intervention with a control group that did not.

The decision to focus on educational measures stems from prior findings suggesting that awareness and understanding of academic ethics can improve student behavior (Chankova, 2020; Stone, 2023). Through direct engagement with the principles of integrity, students may internalize values that promote ethical academic conduct and reject dishonesty. Furthermore, the study acknowledges that academic integrity is not only a behavioral issue but also a philosophical one. Scholars have long debated the nature of truth and honesty—concepts central to the academic mission. While Western ethical theories, such as deontology and utilitarianism, offer different perspectives on integrity, these frameworks may not always translate seamlessly across cultural contexts (Chen & Macfarlane, 2024; Du, 2018; McKenzie, 2018). Despite these complexities, there is a growing consensus that cultivating a culture of integrity is essential for preparing students for ethical decision-making in professional and civic life (Poff, 2023). Therefore, this study contributes to the broader discourse on academic ethics by empirically testing whether targeted educational interventions can foster such a culture. In summary, this article aims to demonstrate how structured academic integrity programs can serve as an effective tool for reducing dishonest behavior and promoting ethical awareness among students in Chinese higher education institutions.

### Academic integrity: theoretical foundations and the chinese context

Academic integrity comprises a set of ethical principles that guide the conduct of students and educators in the academic environment. Its core components include honesty, trust, fairness, respect, and responsibility (Cotton et al., 2024). These values form the cornerstone of university culture, yet their interpretation and implementation depend on cultural, institutional, and technological factors. Research indicates that tolerance for academic misconduct varies across cultural contexts. In individualistic cultures, personal accountability tends to be emphasized, whereas in collectivist societies such as China, violations may sometimes be justified for the sake of collective benefit (Almutairi, 2022; McKenzie, 2018; Striepe et al., 2023). This necessitates the development of culturally sensitive approaches to fostering academic integrity.

Globally, numerous educational efforts have been undertaken to promote a culture of academic integrity. In Canada, Australia, the United Kingdom, and the Baltic countries, these programs have incorporated online courses, seminars, role-playing activities, and ethical dilemma discussions (Anohina-Naumeca et al., 2020; Benson et al., 2019; Slade & Benson, 2020; Sotiriadou et al., 2020; Stoesz et al., 2019). These initiatives have not only raised student awareness but also actively engaged them in the process of developing academic ethics, thereby enhancing the effectiveness of integrity policies. The successful implementation of such programs depends crucially on faculty and administrative support, as well as the existence of clear sanction mechanisms (Chankova, 2020; Cronan et al., 2018; Foltýnek & Dlabolová, 2020; Kornytska, 2023). The advancement of digital technologies, including artificial intelligence, has introduced both risks and new tools for maintaining academic integrity. On the one hand, students have gained more opportunities for cheating and fabrication (e.g., through ChatGPT), while on the other hand, universities have increasingly adopted plagiarism detection systems and digital platforms for monitoring violations (Cotton et al., 2024; Glendinning, 2022; Iliichuk, 2021; Nazarov, 2020).

The Chinese context presents unique challenges regarding academic integrity. As noted by some scholars (Cao et al., 2023), many Chinese universities lack clearly defined academic integrity policies, leaving students poorly informed about acceptable and unacceptable behaviors, while sanctions are inconsistently enforced. As highlighted in another study (Du, 2018), the pressure to achieve high grades, heavy examination loads, and intense competition often lead students to perceive dishonest practices as justifiable. Furthermore, Ma and Shi (2024) emphasize that bureaucratic institutional structures and the absence of robust support mechanisms may negatively affect academic ethics in China. While international best practices in academic integrity exist, their implementation in China requires careful adaptation. Research indicates that previous interventions have been predominantly declarative in nature. Such interventions are often limited to formal measures and fail to offer sustainable and functional implementation mechanisms (Cao et al., 2023). The specific characteristics of the Chinese academic context necessitate adjustments to international approaches. Particular attention should be paid to underdeveloped professional self-regulation and the strong influence of hierarchical structures (Chen & Macfarlane, 2024). The Chinese education system, which emphasizes discipline and rote memorization, rarely incorporates discussions on ethical dilemmas or encourages critical thinking (Chen & Macfarlane, 2024). Consequently, empirical research is needed to assess the effectiveness of educational interventions tailored to the cultural and institutional context of Chinese higher education. This study aims to address this gap by evaluating the impact of a targeted educational program on the attitudes and behaviors of undergraduate students in China.

### Problem statement

China’s higher education system undergoes active transformation through internationalization, digitalization, and increasing academic competition. Therefore, the issue of academic dishonesty has gained particular significance. Growing pressure on students stemming from rigorous academic demands and career competition has contributed to the proliferation of unethical practices. Research indicates that between 15.4% and 51.7% of Chinese university students admit to engaging in some form of academic misconduct (Liu & Alias, 2023). More than two-thirds of surveyed students reported having committed at least one violation during the previous academic year, whether during examinations or when completing assignments. Despite the scale of the problem, China still lacks targeted educational interventions designed to foster ethical student behavior. Furthermore, current literature largely centers on the phenomenon itself, with limited attention to practical solutions. The present study endeavors to respond to this gap. Undergraduate students were selected as the focus of analysis to address the critical period when enduring ethical attitudes are formed, and violations can have long-term consequences for both the academic environment and future professional conduct (Eshet, 2024; Tian & Tang, 2025). The study’s novelty lies in its empirical evaluation of a comprehensive educational program aimed at strengthening academic integrity, while accounting for the cultural and institutional context of Chinese universities. The main purpose of the study is to evaluate the effectiveness of targeted educational programs aimed at improving academic integrity among Chinese university students. To achieve this goal, it is necessary to accomplish the following objectives:

1. Conduct a baseline study to assess existing attitudes toward academic integrity among students.
2. Implement a series of educational activities specifically designed to address the problem of academic dishonesty.
3. Compare the data before and after the intervention to assess the effectiveness of implemented educational strategies.
4. Draw conclusions on the role of educational programs in improving academic integrity.

By defining effective strategies, educational institutions can improve their education quality and reputation. In addition, the results are expected to provide insight into how educational activities can be tailored to the cultural and systemic context, presenting a model for other regions with similar problems. The article aims to contribute to the broader discussion of educational ethics by offering an analysis of how targeted educational interventions promote academic integrity in higher education.

## Research methods

### Instrument

The scale of academic dishonesty developed by Rawwas and Isakson measures four factors related to academic dishonesty (Rawwas & Isakson, 2000). The first subscale, *Acceptance of Cheating*, focuses on direct participation in dishonest behavior such as cheating during exams, helping others cheat, or committing plagiarism. *Acceptance of Cheating* includes six items, which can be rated on a scale from 1 (never) to 5 (always). The total possible score for this parameter varies from 6 to 30, where higher scores indicate a greater propensity for dishonest behavior. The second subscale, *Unfair Advantage*, measures the extent to which students gain advantages in undeserved ways, such as favoritism from teachers or undue advantage because of their status. The scale for this factor includes six items evaluated on a 5-point scale, with a maximum score of 30. The third subscale, *Fabrication*, evaluates behavior related to the fabrication of data or sources, which is crucial for tasks such as writing articles or conducting research. Similarly, the scale also consists of six items, which can be evaluated on a scale from 1 to 5, with a total maximum score of 30. Finally, *Disregard for Dishonest Practices* considers adopting common practices that may be ethically questionable, such as ignoring dishonest actions. The scale for this factor consists of four items with possible scores from 4 to 20. Together, these measures provide a comprehensive assessment of various aspects of academic dishonesty, allowing for an analysis of how educational interventions can affect student attitudes and behaviors regarding academic integrity.

### Instrument validation

To assess the four dimensions of academic misconduct (acceptance of cheating, unfair advantage, fabrication, and disregard for dishonest practices), the study employed a scale originally developed by Rawwas and Isakson (2000) and subsequently adapted for the Chinese context (Rawwas et al., 2004). This instrument has previously demonstrated adequate psychometric properties in this context. A pilot test involving 92 students was conducted to preliminarily validate the instrument. The reliability of each subscale was examined using Cronbach’s alpha, composite reliability (CR), McDonald’s omega (Ω), and average variance extracted (AVE). These measures allowed for a thorough evaluation of the instrument’s consistency and internal reliability. The results indicated high reliability across all subscales: α = 0.89, CR = 0.92, Ω = 0.90, AVE = 0.64 for *Acceptance of Cheating*; α = 0.87, CR = 0.88, Ω = 0.88, AVE = 0.62 for *Unfair Advantage*; α = 0.86, CR = 0.88, Ω = 0.87, AVE = 0.59 for *Fabrication*; α = 0.82, CR = 0.84, Ω = 0.83, AVE = 0.56 for *Disregard for Dishonest Practices*. Confirmatory factor analysis (CFA) demonstrated a good model fit (χ^2^/df = 2.11; CFI = 0.961; TLI = 0.947; RMSEA = 0.053), confirming the construct validity of the scales. These findings indicate that the adapted instrument possesses a robust structure and is suitable for use in Chinese academic settings.

### Intervention

The educational program for the current intervention was developed using principles of constructivist pedagogy and a cognitive-behavioral approach. These frameworks are known to foster enduring student dispositions through practice and active participation (Beck, 2011; Biggs, 1996; Vygotsky, 1978). Elements of Bandura’s social learning theory were also incorporated to create a social environment and enable behavioral modeling (Bandura & National Institute of Mental Health, 1986). Within the Chinese context, the integration of constructivist and cognitive-behavioral principles was anticipated to promote critical thinking and enhance the capacity for sound and ethical decision-making. This is particularly relevant for students developing within an atmosphere dominated by hierarchy and where rote memorization is often the primary learning principle. The intervention was conducted in an in-person format on the premises of the university. All activities were integrated into the regular academic schedule. Sessions were led by faculty members from the Department of Pedagogy and Psychology who had received prior training in academic ethics. Additionally, external experts, including practicing educators and specialists in educational ethics, participated in selected lectures. This facilitated a multidisciplinary approach and enhanced the relevance of the presented material.

The program duration was six months. This period was sufficient to ensure gradual material assimilation, attitude reinforcement, and observation of sustained changes in attitudes toward academic integrity. Each month focused on a specific thematic area and the corresponding behavioral aspect reflected in the scale of academic dishonesty: cheating acceptance, unfair advantage, fabrication, and disregard for dishonest practices. The first month introduced students to the concept of academic integrity, presenting both international and local standards along with examples of violations. During the second and third months, participants engaged in interactive seminars, lectures delivered by invited experts, and case study analyses designed to develop critical thinking skills and the ability to recognize various forms of misconduct. Special emphasis was placed on discussing scenarios involving data fabrication, privilege abuse, cheating, and tacit acceptance of violations. The fourth month featured role-playing exercises and scenario simulations that replicated typical academic situations. These activities allowed participants to practice ethical decision-making. The fifth month involved collecting student feedback and facilitating self-reflection regarding personal attitudes and behavioral changes. During the final stage, participants formulated personal commitments to uphold academic integrity and engaged in group discussions about the importance of ethical standards in academic and professional life. Table 1 provides an overview of the main activities, objectives, and materials used each month to achieve the goals of the intervention.

**Table 1:** Six-month program for promoting academic integrity among Chinese students

Month	Key Activities	Objectives	Materials Used
Month 1: Introduction	Introduction to Academic Integrity, Initial Awareness Campaign	To introduce the concept of academic integrity and assess initial student understanding	Presentation slides, introductory videos, initial assessment surveys
Months 2–3: Education & Engagement	Interactive Workshops, Guest Lectures, Group Discussions	To educate students on the nuances of academic honesty and engage them through discussions and expert insights	Workshop materials, expert talks, discussion guides
Month 4: Practical Application	Role-playing exercises, Scenario Analysis	To apply learned concepts in simulated environments to enhance practical understanding	Scenarios for role-play, analysis tools, feedback forms
Month 5: Evaluation & Feedback	Mid-program Surveys, Feedback Collection	To evaluate the effectiveness of the program at the current stage and integrate student feedback	Survey instruments, feedback collection forms
Month 6: Integration and Ongoing Commitment	Consolidation Workshop, Reflective Discussions, Ongoing Commitment Pledge	To consolidate learning, reflect on the program’s impact	Interactive workshop guides, discussion outlines

The program’s structure was specifically designed to modify the behavioral and cognitive aspects measured during pre-test and post-test evaluations. Each program component corresponded to a specific factor: knowledge and rejection of direct cheating, criticism of privileged positions, unacceptability of fabrication, and the development of the ability to speak up against dishonesty. This multi-level structure enabled targeted influence on student attitudes and contributed to sustainable behavioral changes.

### Participants

Participants represented a wide range of academic disciplines at [BLINDED] School, including humanities, natural sciences, engineering, and economics. Most students were in their second or fourth year of study, encompassing both initial and more advanced stages of academic experience. The study involved a total of 400 undergraduate students, providing a reliable sample size for statistical analysis. Participants were selected through stratified random sampling to maintain diversity in terms of gender, age, and academic major. Following selection, they were randomly assigned to either the experimental or the control group. The randomization procedure employed computer-generated sequences to eliminate selection bias. There was an almost equal gender distribution in the sample: 202 male students (51%) and 198 female students (49%). The age of the students varied between 18 and 25, with an average age of 21. No participant attrition occurred during the study. All students included in the sample completed both the pre-test and post-test, ensuring data integrity.

### Research design

The scale of academic dishonesty served as the evaluation instrument, administered both during pre-testing and program completion (post-test). Preliminary data collection occurred in October 2024 on university premises, with final testing conducted in April 2025 at the same location immediately following the six-month educational program. This design ensured that any observed intergroup differences could be directly attributed to the educational intervention, as all other variables remained constant. For six months, the experimental group participated in various structured activities of the academic integrity program. On the other hand, the control group did not participate in any activities but followed their standard curriculum, which did not focus on topics of academic integrity. This approach suggests that any observed differences in attitudes towards academic integrity between the control and experimental groups are directly related to the impact of the program. The setting described above provides a basis for evaluating the effectiveness of educational activities aimed at promoting ethical academic practices.

### Ethical issues

Ethical aspects were of paramount importance for this study to ensure the protection and respect of all participants. Before starting the study, the authors obtained the approval of the Ethics Council at [BLINDED] School. All participants received detailed information about the study. Each participant provided informed consent, which stated the right to withdraw from the study at any time without any consequences. To preserve confidentiality, all personal data was depersonalized before the analysis. No individual identifiers were used in any reports or publications. In addition, measures were taken to ensure that all information was securely stored and accessible only to the research team. These measures ensured compliance with international standards for the ethical conduct of research.

### Data analysis

For data analysis, the study employed the statistical software SPSS 26. The data analysis for this study involved using two main statistical methods: descriptive statistics and t-tests. Descriptive statistics summarized the main characteristics of the study data, providing simple summaries of the sample and indicators. In turn, t-tests were used to evaluate the effectiveness of the academic integrity program. In the experimental group, a paired t-test was used to compare the pre-test and post-test results. These tests showed whether the intervention led to significant changes in students' scores on the scale of academic dishonesty, indicating a decrease in the propensity for academic dishonesty. To compare the experimental and control groups, a t-test of independent samples was performed to analyze the differences in changes between the two groups from the pre-test to the post-test. This analysis was crucial for understanding whether the changes observed in the experimental group were statistically significant compared to the control group, which did not receive any target program. These methods served as the basis for evaluating the hypothesis that the academic integrity program significantly influences students' attitudes and behavior toward academic dishonesty. The significance level was set at *p* < 0.05 for all tests to ensure reliability and statistically significant results. The data distribution was normal.

## Results

Table 2 presents descriptive statistics and t-test results for the control and experimental groups.

**Table 2:** Descriptive statistics of academic dishonesty scores before and after intervention for control and experimental groups

Group		Acceptance of Cheating Scale_pre-test	Unfair_Advantage_pre-test	Fabrication_pre-test	Disregard for Dishonest Practices_Practices_pre-test	Acceptance of Cheating Scale_post-test	Unfair_Advantage_post-test	Fabrication_post-test	Disregard for Dishonest Practices_ post-test
Control group	Mean	13.48	14.59	14.71	11.75	13.48	14.59	14.71	11.75
	N	200	200	200	200	200	200	200	200
	Standard deviation	4.977	5.985	5.300	4.949	4.977	5.985	5.300	4.949
Experimental group	Mean	13.54	15.28	14.83	11.67	11.08*	12.80*	12.37*	14.19*
	N	200	200	200	200	200	200	200	200
	Standard deviation	4.942	5.406	5.163	4.840	5.011	5.511	5.316	4.969

Statistically significant differences in the post-test are marked with *p <.001

In the control group, which did not participate in the program, mean scores across all four subscales of academic dishonesty—*Acceptance of Cheating*, *Unfair Advantage*, *Fabrication*, and *Disregard for Dishonest Practices*—remained statistically stable between pre-test and post-test measurements. Specifically, the subscale *Acceptance of cheating* showed no change (13.48 in both pre-test and post-test; t(199) = −1.202, *p* = 0.231). The subscale *Unfair Advantage* exhibited a marginal increase (from 14.59 to 14.85) that did not reach statistical significance (t(199) = 1.859, *p* = 0.065). *Fabrication* demonstrated a slight decrease (from 14.71 to 14.56), which was also non-significant (t(199) = −1.053, *p* = 0.293). The subscale *Disregard for Dishonest Practices* showed a minor increase (from 11.75 to 11.97), but this difference was not statistically significant (t(199) = 1.577, *p* = 0.116). These results indicate no considerable changes in the attitudes of students in the control group, confirming measurement stability and the absence of external variable influence during the observation period.

In contrast, the experimental group showed marked positive changes across all scales after completing the six-month academic integrity training program. The mean score for *Acceptance of cheating* decreased from 13.54 to 11.08 (t(199) = 4.816, *p* < 0.001). The scores on the subscale *Unfair Advantage* declined from 15.28 to 12.80 (t(199) = 3.120, *p* = 0.002). Similarly, *Fabrication* scores decreased from 14.83 to 12.37 (t(199) = 4.399, *p* < 0.001). Conversely, the scores on the subscale *Disregard for dishonest practices* increased from 11.67 to 14.19 (t(199) = −4.931, *p* < 0.001). This indicates enhanced student sensitivity to academic ethics violations and decreased tolerance for dishonest behavior. All differences between pre-test and post-test results in the experimental group were statistically significant, demonstrating a clear intervention effect.

Preliminary equivalence testing using Levene’s test confirmed variance homogeneity across all scales, except for *Unfair Advantage*, which required adjustment for unequal variances. Post-intervention comparisons between the groups revealed significant differences on all scales (*p* < 0.001), confirming the program’s effectiveness in fostering academic integrity. In general, the results demonstrate that the educational intervention had a significant positive impact on students’ behavioral attitudes toward academic integrity. The program effectively reduced tendencies toward dishonest behavior while increasing awareness and intolerance of academic norm violations.

## Discussion

The findings of the present study suggest that participation in an educational program focused on academic integrity leads to reduced student propensity for dishonest behaviors and increased intolerance toward ethical violations. Unlike the control group, which showed statistically insignificant changes across all scales (*p* >.05), the experimental group demonstrated marked positive improvements. These results indicate the potential impact of several factors on the intervention’s effectiveness. One possible explanation lies in the program’s cognitive-behavioral foundation, which systematically develops ethical beliefs and skills through reflective discussions, role modeling, and feedback. As suggested by Chen and Macfarlane (2024), a structured, multi-level approach may enhance intrinsic motivation to adhere to academic norms. This could account for the observed changes in attitudes regarding cheating acceptance, fabrication, and unfair advantage. The intervention progressively engaged students in active learning through discussions, role-playing, and case analyses, which may have reinforced normative behaviors. These conclusions align with observations by Sotiriadou et al. (2020), who emphasize that long-term programs incorporating practice-oriented components can yield lasting behavioral changes, particularly in ethical domains.

The program was specifically adapted to the Chinese sociocultural context. Research (Cao et al., 2023) indicates that in China, academic integrity norms may be perceived as externally imposed, with students often lacking a clear understanding of acceptable boundaries. To address this, the intervention incorporated discussions of specific ethical dilemmas (ignoring plagiarism by classmates, data fabrication, cheating on exams, copying homework, requests to include non-contributing members in projects, improper use of artificial intelligence), potentially increasing student awareness and reducing the “normalization” of dishonest behavior. This suggestion corroborates the findings by Denisova-Schmidt (2018), underscoring the importance of cultural sensitivity and clear institutional signals in promoting academic integrity. The program’s emphasis on student co-authorship (actively involving participants in developing shared values and rules) may have fostered subjective engagement and personal accountability. As demonstrated by Ayodele et al. (2019), programs that engage students in the development of integrity policies can bridge the gap between formal standards and daily practice. Consequently, such programs facilitate the acceptance of academic ethics.

The intervention described in this study also addressed challenges posed by the digital era. As noted by Cotton et al. (2024), the use of AI tools like ChatGPT introduces new risks to academic integrity. Incorporating discussions about ethics in digital environments likely enhanced the program’s relevance and fostered students’ reflective attitudes toward technology. Similarly, employing automated misconduct detection tools, as described by Glendinning (2022), may reinforce the perception of academic rules as meaningful and traceable. Additionally, the proposed program implemented a multi-level strategy involving faculty and experts. This approach is supported by the literature—Almutairi (2022) demonstrates that the behavior of teachers as role models substantially influences the behavior of students. The participation of external experts and faculty in program delivery may have provided additional legitimacy to the discussed values. Certain elements of the program, including gamification components and student code development, likely increased engagement. Khan et al. (2021) show that game-based and interactive approaches promote better norm internalization and behavioral persistence. Finally, the idea of sustainable development in education may serve as evidence of the program’s effectiveness. As Kudeikina et al. (2022) demonstrate, academic integrity is not merely a short-term goal but a sustainability strategy for educational environments. These findings suggest that ethics-focused programs may establish foundations for long-term institutional resilience and enhanced trust in educational systems.

Several limitations and future research directions warrant discussion. The study was conducted with students from a single university, limiting the generalizability of the findings to other Chinese institutions or international contexts. Educational conditions, policies on academic integrity, and cultural norms vary substantially across regions, potentially affecting the applicability of the model elsewhere. The intervention and observation period lasted six months. While statistically significant changes in student attitudes were detected, the long-term sustainability of these effects remains uncertain. Future studies could incorporate follow-up measurements (after one year or more) to assess whether changes persist over time. The assessment of academic integrity relied solely on participant self-reports, which may be subject to distortions from social desirability bias or underreporting of personal conduct. The study did not include behavioral indicators that could verify actual behavioral changes, such as the number of disciplinary violations, analysis of plagiarism detection reports, or analytical data from learning management systems. Future research should employ objective behavioral measures alongside survey data, potentially through observations, case analyses, and institutional statistics. Additionally, it is necessary to investigate how individual and contextual factors—including academic motivation, field of study, gender, and prior history of norm violations—affect student responsiveness to such programs. This could enhance the effectiveness of integrity programs through more precise adaptations for different student groups.

## Conclusions

The study results demonstrate statistically significant reductions in academic misconduct among students in the experimental group following their participation in academic integrity training. Specifically, mean scores decreased from 13.54 to 11.08 (*p* < 0.001) on the subscale *Acceptance of Cheating*, from 15.28 to 12.80 (*p* < 0.001) on *Unfair Advantage*, and from 14.83 to 12.37 (p < 0.001) on *Fabrication*. Concurrently, sensitivity to unethical behavior increased significantly, with mean scores on the subscale *Disregard for Dishonest Practices* rising from 11.67 to 14.19 (*p* < 0.001). These findings underscore the effectiveness of the six-month intervention in fostering students’ conscious engagement with academic and ethical norms. The program appears to facilitate a deeper understanding of the consequences that follow from misconduct, potentially improving academic outcomes and establishing sustainable behavioral changes. Integrating integrity discussions into curricula allows students to connect these values with future professional practice, particularly in fields like medicine, law, engineering, and education, where ethical standards prove critical. The conclusions indicate the need for an expanded implementation of academic integrity programs adapted to diverse cultural and educational contexts. Future research should assess the long-term sustainability of the observed effects and examine faculty involvement in such initiatives. This systemic approach can lay the groundwork for the promotion of academic integrity as a key pillar of institutional culture and broader social responsibility.

## Data Availability

All data generated or analysed during this study are included in this published article.
